# Resected intramuscular hemangioma in the chest wall: a case report

**DOI:** 10.1186/s40792-024-02023-4

**Published:** 2024-09-26

**Authors:** Yoshiyuki Nakanishi, Takaki Akamine, Fumihiko Kinoshita, Mikihiro Kohno, Keigo Ozono, Takuya Hino, Taro Mori, Yoshinao Oda, Tomoyoshi Takenaka, Masafumi Nakamura

**Affiliations:** 1 Department of Thoracic Surgery, Kyushu University Hospital, 3-1-1, Maidashi, Higashi-Ku, Fukuoka, 812-8582 Japan; 2 Department of Surgery and Oncology, Graduate School of Medical Science, Kyushu University, 3-1-1, Maidashi, Higashi-Ku, Fukuoka, 812-8582 Japan; 3 Department of Surgery and Science, Graduate School of Medical Science, Kyushu University, 3-1-1, Maidashi, Higashi-ku, Fukuoka, 812-8582 Japan; 4 Department of Anatomic Pathology, Pathological Sciences, Graduate School of Medical Science, Kyushu University, 3-1-1, Maidashi, Higashi-Ku, Fukuoka, 812-8582 Japan; 5 Department of Clinical Radiology, Graduate School of Medical Science, Kyushu University, 3-1-1, Maidashi, Higashi-Ku, Fukuoka, 812-8582 Japan

## Abstract

**Background:**

Intramuscular hemangioma is an uncommon benign tumor found mainly in the limbs of adolescents and young adults. The local recurrence rate is high, ranging from 30 to 50%, necessitating wide local excision of intercostal intramuscular hemangiomas. However, preoperative diagnosis of intramuscular hemangiomas is challenging. Herein, we report a rare case of an intramuscular hemangioma arising from the chest wall.

**Case presentation:**

A healthy 29-year-old asymptomatic man was referred to our hospital after an abnormal shadow was observed on his chest radiography. Computed tomography and magnetic resonance imaging revealed a 30-mm-sized mass in the right second intercostal space. Neoplastic lesions, such as schwannomas or solitary fibrous tumors, were included in the preoperative differential diagnosis. Tumor resection was performed using video-assisted thoracoscopic surgery. The tumor, which had a smooth surface covered with parietal pleura, was dissected from the external intercostal muscle and costal bone. Postoperative histopathological examination revealed proliferation of spindle-shaped endothelial cells arranged in a capillary vascular structure accompanied by entrapped smooth muscle fibers, adipose tissue, and muscle vessels. The final diagnosis was an intramuscular hemangioma with negative surgical margins. There was no evidence of recurrence during the 1-year postoperative follow-up period.

**Conclusion:**

Intramuscular hemangiomas should be considered in the differential diagnosis of chest wall tumors, particularly in young people, owing to their potential for recurrence. Moreover, postoperative follow-up may be necessary for resected intramuscular intercostal hemangiomas.

## Background

Intramuscular hemangioma is a rare benign tumor mainly found in the limbs of adolescents and young adults, often presenting with pain [1, 4]. Hemangioma arising from the chest wall is extremely rare and has only been reported in a few documented cases [2, 4]. Preoperative diagnosis of intramuscular hemangioma is challenging and often relies on postoperative histopathological confirmation. Given its high local recurrence rate (30–50%), resection with enough surgical margin is crucial [3]. We report the case of a 29-year-old man who was incidentally discovered to have a chest wall tumor diagnosed as intercostal intramuscular hemangioma from a specimen resected under video-assisted thoracoscopic surgery (VATS), which was preoperatively unexpected.

## Case presentation

**Fig. 1:**
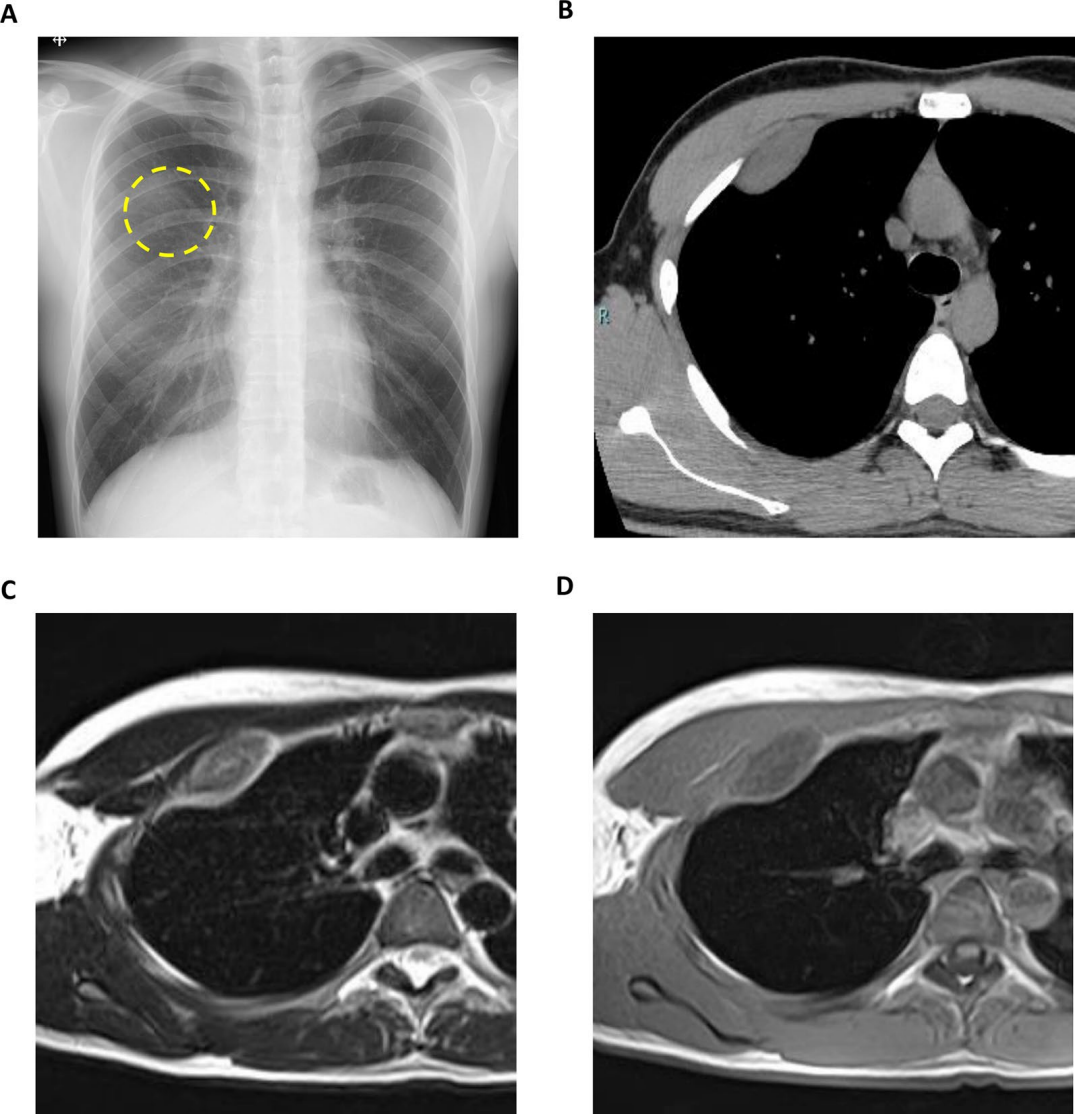
**a** Chest radiography showed an abnormal shadow in the right upper lung field (dotted circle). **b** Chest computed tomography showed a 30-mm mass in the right second intercostal space. **c** T2-weighted MRI scan showed a high-intensity area in the right second intercostal space without evidence of peritumoral invasion. **d** T1-weighted MRI scan showed intermediate intensity area in the right second intercostal space. MRI: Magnetic resonance imaging

**Fig. 2:**
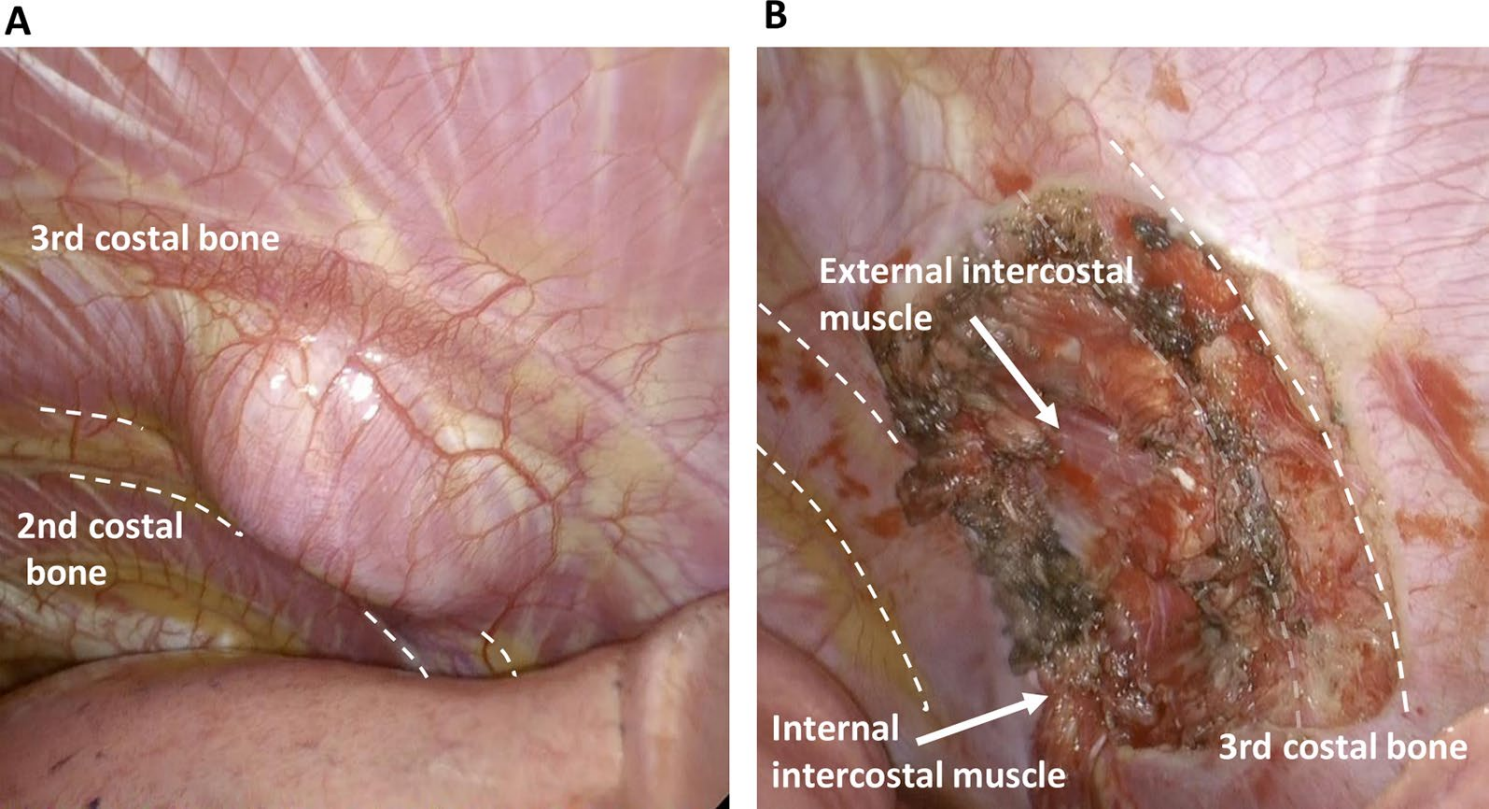
Intraoperative findings. **a** The tumor surface was smooth and covered with pleura. **b** The tumor was dissected from external intercostal muscle and costal bone

**Fig. 3:**
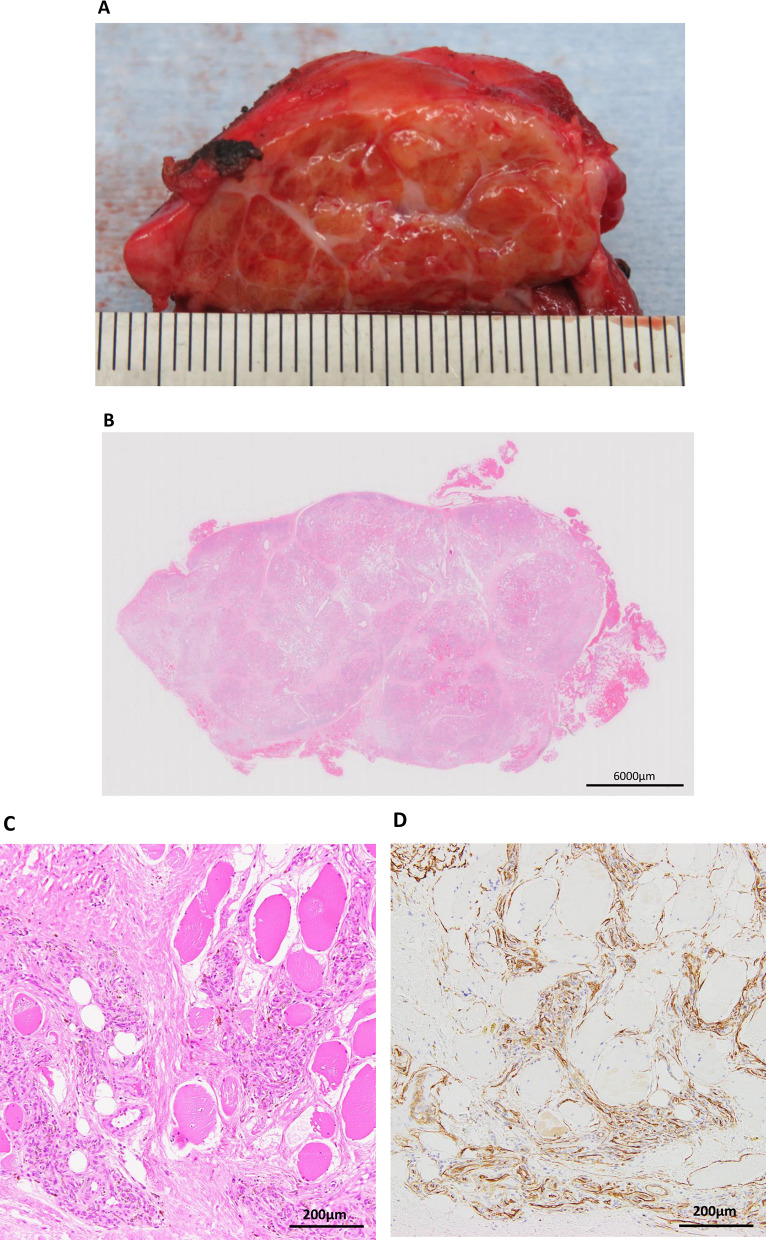
Gross and histopathological findings. **a** The tumor was a well-circumscribed yellowish-white nodule. **b**, **c** Microscopic findings (H&E stain). The tumor was well-demarcated. Smooth muscle fibers were replaced by dilated vascular channels with focal thrombosis and adipose tissue. **d** Endothelial cells are positive for CD34

A healthy 29-year-old man with no relevant medical history presented with an abnormal opacity in the right upper field on chest X-ray screening during a routine health check (Fig. 1a). Blood tests, including those for tumor markers, were normal. Plain computed tomography (CT) revealed a 30-mm mass in the second intercostal space (ICS), that was well-circumscribed and homogeneous (Fig. 1b). Plain magnetic resonance imaging (MRI) showed high intensity on T2-weighted images and intermediate intensity on T1-weighted images, with no sign of infiltration into the surrounding tissue, costal bone, or lungs (Fig. 1c and d). Imaging revealed no signs of hemorrhage or calcification on image examinations. Suspecting a schwannoma or solitary fibrous tumor, we performed VATS for diagnosis and treatment. Under general anesthesia, the patient was placed in the left lateral position and three ports were placed. The tumor was identified on the chest wall anterior to the second ICS and had a smooth surface covered with the parietal pleura and internal intercostal muscle (Fig. 2a). The pleural wall was incised along the tumor margin, and the tumor, located between internal and external intercostal muscles, was dissected from the external intercostal muscle and costal bone (Fig. 2b). Gross examination of the surgical specimen revealed that the tumor was a well-circumscribed yellowish-white nodule (Fig. 3a). Histopathological examination showed proliferation of spindle-shaped endothelial cells arranged in a capillary vascular structure, accompanied by entrapped smooth muscle fibers, adipose tissue, and muscle vessels (Fig. 3b and c). The surgical margin was 2 mm and microscopically negative. Immunohistochemically, the endothelial cells were positive for CD34 (Fig. 3d). These pathological features led to a diagnosis of intramuscular hemangioma. The patient’s postoperative course was uneventful, and he was discharged after 5 days. No evidence of recurrence was found at 12 months after surgery.

## Discussion

Intramuscular hemangioma is defined by the World Health Organization classification as a proliferation of benign vascular channels within skeletal muscle, associated in most instances with variable amounts of mature adipose tissue [4]. The lower limbs are commonly affected, particularly the thigh and calf, followed by the head, neck, and upper limbs, while the tumor rarely arises in the chest wall [4]. Only six cases of resected intercostal intramuscular hemangioma have been reported in the literature (Table 1). Intramuscular hemangioma recurs at a high rate (30–50%) despite the tumor’s benignity [3]. We speculate that intercostal intramuscular hemangiomas also pose a high risk of recurrence. Therefore, regardless of their rarity, these tumors should be included in the differential diagnosis of chest wall tumors, especially in young people.

**Table 1:** Reported cases of resected intercostal intramuscular hemangioma as defined by the World Health Organization classification of soft tissue tumors

Case reports	Age (years)/Sex	Symptoms	Location	Tumor size (cm)	Contrast examination		Preoperative diagnosis	Dumbbell-type tumor	Biopsy	Surgical procedure			Follow-up period (months)	Recurrence
										Rib resection		Reconstruction of chest wall		
					CT	MRI				+ / −	Resected bone			
David, et al., 1992 [5]	39/F	None	Rt. 4–5th ICS	5.0	–	–	Non-diagnosis	No	Yes	+	4th, 5th, 6th ribs	+	N.A	N.A
Ono, et al., 1996 [6]	33/M	None	Rt. 4th ICS	9.5	–	–	N.A	No	No	–		–	18	None
Yonehara, et al., 2000 [7]	33/M	None	Lt. 6th ICS	5.0	–	–	Angiolipoma susp.	Yes	Yes	+	7th rib cartilage	–	30	None
Kubo, et al., 2004 [8]	27/M	Chest pain and shortness of breath after exertion	Rt. 7th ICS	5.5	–	+	Hemangioma susp.	Yes	No	+	7th, 8th ribs	+	6	None
Elbaweb, et al., 2019 [9]	14/M	None	Rt. 5th ICS	6.5	+	+	Hemangioma susp.	Yes	No	+	5th, 6th ribs	+	6	None
Ochi, et al., 2023 [10]	17/F	None	Lt. 2nd ICS	2.9	–	–	Neurogenic tumor susp.	No	No	–		–	18	None
Present case	29/M	None	Rt. 2nd ICS	3.0	–	–	Neurogenic tumor or SFT susp.	No	No	–		–	12	None

ICS, intercostal space; SFT, solitary fibrous tumor; N.A., not available; MRI, magnetic resonance imaging; CT, computed tomography

Clinically, intramuscular hemangiomas often occur in adolescents and young adults, without sex-related differences. Moreover, a previous report has shown that a history of trauma implicates the genesis and growth of intramuscular hemangiomas [7]. The primary symptom of a tumor in the extremities is exercise-induced pain, although only one previously reported case of intercostal intramuscular hemangioma had these symptoms (Table 1) [1, 8]. Hence, the possibility of an intramuscular hemangioma should be considered in asymptomatic chest wall tumors, as in the present case.

Preoperative diagnosis of intercostal intramuscular hemangiomas is challenging (Table 1**)**. Preoperative biopsy did not lead to a correct diagnosis in two previous case reports. In a previously reported case similar to the present case, a neurogenic tumor was also suspected because the plain CT and MRI findings resembled those tumors [10]. Hemorrhage or calcification can sometimes be found in larger hemangiomas on CT and MRI, while neurogenic tumors can present the same in imaging findings, though infrequently. Only two prior cases of dumbbell-shaped tumors with preoperative contrast-enhanced MRI examinations were diagnosed correctly [8, 9]. Dynamic contrast-enhanced MRI findings can be helpful for the diagnosis of hemangiomas, which are reported to have eccentric enhancement with incomplete peripheral filling in the early phase and filling during the delayed phase [9]. Therefore, CT or MRI examinations should be performed with contrast media, even in younger patients.

A retrospective cohort study reported a series of 77 patients with intramuscular hemangiomas who underwent surgical excision over a 25-year period [3]. Most recurrent cases of intramuscular hemangioma showed insufficient surgical margins (21 of 23 cases), suggesting that surgical margin and tumor size are the major determinants for local recurrence in patients with intramuscular hemangioma, indicating the necessity of wide resection with sufficient surgical margins [3]. On the other hand, there are no reports of recurrence in the six previous cases of resected intercostal intramuscular hemangioma (Table 1). Presumably, intercostal hemangiomas grow slowly without infiltration depending on the nature of their benignity. In addition, complete excision was accomplished through rib resection and reconstruction of the chest wall in three previous cases of dumbbell-type tumors arising from the intrathoracic to extrathoracic region, with the ICS as a narrow section [7–9, 11].

In our case, marginal excision was performed only with partial resection of the intercostal muscles because the tumor was considered benign owing to intraoperative findings: well-circumscribed, homogeneous, and non-invasive into the surrounding tissue. Consequently, the surgical margin was 2 mm and quite short, but the tumor was completely resected with a negative microscopic surgical margin. A retrospective cohort study reported that patients with completely resected intramuscular hemangiomas had low recurrence rates, with a 92.7% recurrent-free survival rate in 5 years [3]. As mentioned in the previous study, we considered that the possibility of recurrence in our case was low. Therefore, we determined that additional resection was unnecessary.

The previous study above also demonstrated that approximately 10% of cases showed recurrence within 5 years even with wide and complete resection, indicating the necessity of postoperative follow-up after resection of intramuscular hemangioma [3]. In the six previous cases of intercostal intramuscular hemangioma, the mean postoperative observation period was 1.2 years, which may be insufficient (Table 1). Therefore, postoperative follow-up for 5 years may be necessary for patients with resected intercostal intramuscular hemangiomas, even after complete resection, despite the lack of reported recurrences in these cases.

## Conclusion

Intramuscular hemangiomas should be considered in the differential diagnosis of chest wall tumors using enhanced-contrast media, particularly in young patients. Wide resection with sufficient surgical margins is preferable for intramuscular hemangioma because of its high recurrence rate despite its benignity. Postoperative follow-up may also be necessary after resection.

## Data Availability

The availability of the data used in this case is subject to confirmation by the journal or the authors. For more information on data availability and access procedures, please contact the journal or corresponding author.

## Abbreviations

CT: Computed tomography
MRI: Magnetic resonance imaging
VATS: Video-assisted thoracoscopic surgery
ICS: Intercostal space
